# Alternative splicing responses to salt stress in *Glycyrrhiza uralensis* revealed by global profiling of transcriptome RNA-seq datasets

**DOI:** 10.3389/fgene.2024.1397502

**Published:** 2024-07-09

**Authors:** Hua Yao, Guozhi Li, Zhuanzhuan Gao, Fei Guo, Jianghua Feng, Guanghui Xiao, Haitao Shen, Hongbin Li

**Affiliations:** ^1^ Key Laboratory of Xinjiang Phytomedicine Resource and Utilization of Ministry of Education, Key Laboratory of Oasis Town and Mountain-Basin System Ecology of Xinjiang Production and Construction Corps, College of Life Sciences, Shihezi University, Shihezi, China; ^2^ Zhuhai College of Science and Technology, Zhuhai, China; ^3^ Business School of Xinjiang Normal University, Urumqi, China; ^4^ College of Life Sciences, Shaanxi Normal University, Xi’an, China

**Keywords:** *Glycyrrhiza uralensis*, salt stress response, RNA-seq analysis, splicing regulatory factor, post-transcriptional regulation

## Abstract

Excessive reactive oxygen species stress due to salinity poses a significant threat to the growth of *Glycyrrhiza uralensis* Fisch*.* To adapt to salt stress, *G. uralensis* engages in alternative splicing (AS) to generate a variety of proteins that help it withstand the effects of salt stress. While several studies have investigated the impact of alternative splicing on plants stress responses, the mechanisms by which AS interacts with transcriptional regulation to modulate the salt stress response in *G. uralensis* remain poorly understood. In this study, we utilized high-throughput RNA sequencing data to perform a comprehensive analysis of AS events at various time points in *G*. *uralensis* under salt stress, with exon skipping (SE) being the predominant AS type. KEGG enrichment analysis was performed on the different splicing genes (DSG), and pathways associated with AS were significantly enriched, including RNA transport, mRNA surveillance, and spliceosome. This indicated splicing regulation of genes, resulting in AS events under salt stress conditions. Moreover, plant response to salt stress pathways were also enriched, such as mitogen-activated protein kinase signaling pathway – plant, flavonoid biosynthesis, and oxidative phosphorylation. We focused on four differentially significant genes in the MAPK pathway by AS and qRT-PCR analysis. The alternative splicing type of *MPK4* and *SnRK2* was skipped exon (SE). *ETR2* and *RbohD* were retained intron (RI) and alternative 5’splice site (A5SS), respectively. The expression levels of isoform1 of these four genes displayed different but significant increases in different tissue sites and salt stress treatment times. These findings suggest that *MPK4*, *SnRK2*, *ETR2*, and *RbohD* in *G. uralensis* activate the expression of isoform1, leading to the production of more isoform1 protein and thereby enhancing resistance to salt stress. These findings suggest that salt-responsive AS directly and indirectly governs *G. uralensis* salt response. Further investigations into AS function and mechanism during abiotic stresses may offer novel references for bolstering plant stress tolerance.

## 1 Introduction

Increased emissions of greenhouse gases have led to global warming, while high evapotranspiration rates have exacerbated soil salinization (Cicek et al., 2022). A lack of annual rainfall is also preventing salt from reaching the subsoil, resulting in serious ecological changes to soil (Khosravichenar et al., 2023). Xinjiang has a large area of saline-alkali land as a result of insufficient annual precipitation. In recent times, *Glycyrrhiza uralensis* Fisch, widely cultivated in Northwest China for medicinal and food purposes, has suffering from salt exposure throughout the year. For plants to prevent ROS proliferation, the antioxidant defense system must be activated. The SOS pathway, for instance, is activated by salt stress, which results in ROS accumulation and MAPK cascades (Yang et al., 2018).

Plants respond to salt stress through mitogen-activated protein kinase (MAPK) cascades. Three primary components comprise these cascades, each of which is involved in phosphorylation and activation in succession. In order for the second element to function, a MAP kinase (*MAPKK* or *MKK*) must be phosphorylated and activated by the first. As a result of the sequential phosphorylation process, the stress signal can be amplified, thus allowing for a more robust response. Salt-induced stress requires MAPK cascades to transduce signals and enable plants to adapt it (Mishra et al., 2006). The salt stress response of *Arabidopsis* is mediated by the *MPK4* cascade. Null mutants of the gene exhibit hypersensitivity, while overexpression increases salt tolerance (Teige et al., 2004). Other plants also activate the MAPK cascade under salt stress; for example, alfalfa (*Medicago sativa*) activates the MAPK cascade under salt stress (Kiegerl et al., 2000). In rice, the *MPK4* cascade regulates transcription factor gene expression in response to salt stress (F. Wang et al., 2014). When *Arabidopsis* is dehydrated, mutants of the *MKK4* cascade are more salt sensitive and shed more water than the wild type (Kim et al., 2012).

When *G. uralensis* is exposed to salt stress, proteins that are involved in the synthesis of glycyrrhizic acid and liqueritin are activated, resulting in the cumulative production of glycyrrhizin and its components (C. Wang et al., 2020). Alternative splicing events can involve five different forms: skipped exon (SE), alternate 5-splice site (A5SS), alternate 3-splice site (A3SS), mutually exclusion exon (MXE), and retained intron (RI). Some AS events may be gene-specific and some species-specific, with SE and RI being the most and least prevalent, respectively, in animals. The majority of AS cases are caused by SE, while only 0.01% are caused by RI; in contrast, RI is the most common form of AS in *Arabidopsis* and maize (Daszkowska-Golec et al., 2017; Laloum et al., 2018; Thatcher et al., 2016). However, in recent years, SE and RI are the most prevalent AS in licorice and soybeans because of advances in sequencing technology and detection tools (Li et al., 2022; Song et al., 2020). The number of genes with introns that undergo alternative splicing in *Arabidopsis thaliana* (Marquez et al., 2012) will increase as more transcriptome data from plants in different developmental conditions and environmental conditions are collected. High-throughput analyses are becoming more sophisticated as more advanced tools are developed for splice variant identification.

Multiple studies have illustrated the important functions of alternative splicing in the cellular response to abiotic stresses—specifically, salt stress (Laloum et al., 2018). Recent research provides evidence that highlights the essential biological function of alternative splicing (AS) in increasing plant resilience to salt stress. These events have been implicated in regulating stress responses in plants. Spliceosomal components of *Arabidopsis* also affect response to stress in plants. There are over 6,000 *Arabidopsis* genes, for instance, that alter their alternative splicing patterns when exposed to salt stress (Feng et al., 2015). Mutations in *SKIP* protein interact physically with *SR45*, which modulates recognition or cleavage of splice sites 5′ and 3′ in alternative splicing in SNW/Ski-interacting protein (SKIP) (Wang et al., 2012), leading to increased salt and osmotic sensitivity in plants (Feng et al., 2015). In rice, *OsNHX1*-related gene undergoes AS under the salt stress, yielding three distinct transcripts. Notably, the transcript carrying the 3′UTR has been demonstrated to boost salt endurance the most (Amin et al., 2016). Three transcript isoforms of *WDREB2* were produced by AS in wheat: *WDREB2α*, *WDREB2β*, and *WDREB2γ*. During a 24-hour stress treatment period, the expression of the non-functional isoform *WDREB2β* lacking the third exon remained relatively constant. In contrast, the functional isoforms *WDREB2α* and *WDREB2γ* exhibited temporary increases in transcript levels when subjected to drought, salt, and ABA stress treatment (Egawa et al., 2006; Liu et al., 2018).

Recent studies have demonstrated that alternative splicing plays a regulatory role in plants under salt stress (Cinatl et al., 2003). However, studies on how AS responds to salt in *G*. *uralensis* are rarely reported. Therefore, this study aims to investigate the regulatory mechanism of AS and expression profiles of different AS isoforms in response to salt stress by transcriptional regulation in *G*. *uralensis*. To gain valuable insights into the mechanisms underlying salt stress response in *G. uralensis*, we investigated the regulation of alternative splicing (AS) and expression patterns of various AS isoforms. Moreover, we aimed to examine the impact of AS on the development of *G. uralensis* under salt stress. In this study, we conducted RNA sequencing (RNA-seq) experiments on both aboveground parts (AP) and underground parts (UP) of *G. uralensis*. The plants were exposed to salt stress for durations of 0, 2, 6, and 12 h. We identified isoforms and quantified events of alternative splicing at diverse stages of the salt stress. Five AS types were identified at the AP and UP of salt-stressed *G. uralensis*. We identified differentially spliced genes (DSGs) that were then subjected to Gene Ontology (GO) term and Kyoto Encyclopedia of Genes and Genomes (KEGG) pathway analysis. The predominant AS events observed in our study were exon skipping (SE). In *G. uralensis,* we also identified several new AS events that greatly contributed to the response process to salt stress. Our results provide valuable insights into the stress response mechanism in *G*. *uralensis*. The findings illuminate the post-transcriptional regulation patterns of AS at different time points throughout the salt stress process.

## 2 Materials and methods

### 2.1 Plant material and salt stress treatments

The experimental materials were sourced from wild *G. uralensis* plants (Ding et al., 2014). Wild *G. uralensis* seeds were treated with 95% concentrated H_2_SO_4_ for 60 min and rinsed 8–10 times with sterile water and were then let dry. Later, these seeds were placed in a flowerpot containing nutrient soil and vermiculite 1:1. These flowerpots were allowed to germinate at 25°C in an illumination incubator (*GXZ-430D*) under dark conditions before being grown in a climate-controlled chamber. Licorice seedlings 60 days after emergence were transplanted into a hydroponic box (38 × 28 × 14 cm) with nutrient solution (Hogrland nutrient solution) for 5 days to adapt to the hydroponic conditions. NaCl was added to the hydroponic culture solution to a concentration of 150 mM/L. The culture media was changed once every 2 days. After 2, 6, and 12 h of NaCl stress, tissue samples of NaCl treatment for different times were collected. All samples were collected immediately, washed with distilled water, dried, frozen in liquid nitrogen, and stored at −80°C in three biological replicates of each treatment.

### 2.2 RNA-seq datasets and processing methods

The experiment involved all AS in the RNA-seq datasets which were detected by rMATs [(version 4.0.1) (http://rnaseq-mats.sourceforge.net/index.html)]. This incorporated data from 24 libraries, each representing different conditions (two tissue types × time points of salt stress treatment × three biological replicates).

### 2.3 Reference genome-based assembly of transcript structures, mapping reads

A stringent quality control procedure, which can ensure the accuracy of rMATs analysis results, was performed on the RNA-seq raw read datasets. Attaining high-quality raw sequencing data necessitates the elimination of low-quality reads, which contain sequences with high “N” and short reads. To screen out low-quality bases, SeqPrep (https://github.com/jstjohn/SeqPrep) and Sickle (https://github.com/najoshi/sickle) were used (Li et al., 2022). We used the reference genome for the species name: *Glycyrrhiza_uralensis* Reference genome Version: riken (Reference Genome Source: http://ngs-data-archive.psc.riken.jp/Gur-genome/index.pl). We employed TopHat2 (http://ccb.jhu.edu/software/tophat/index.shtml) to locate new splice sites mapped directly to pre-existing transcripts for accurate comparison. In addition, we used String Tie (http://ccb.jhu.edu/software/stringtie/) to assemble complex datasets into transcripts and compared them with known transcripts.

### 2.4 AS events identification in response to salt stress

We used rMATs to identify and analyze AS events between these samples. We detected AS events showing a significant salt stress response by comparing them to AS events with a false discovery rate (FDR) < 0.01. We identified five AS event types: SE, MXE, A5SS, A3SS, and RI.

### 2.5 DEGs, gene ontology, and pathway enrichment analysis

We used DESeq2V (http://bioconductor.org/packages/stats/bioc/DESeq2/) to analyze RNA-seq datasets in pair comparisons between the salt-stressed treatment and control group to identify DEGs. We identified genes as differentially expressed (DEGs) when their fold change was greater than or equal to 2 and their false discovery rate (FDR) was below 0.05. Transcripts per million (TPM) were utilized to quantify the gene expression levels in our study. DSGs were determined based on the FDR (*p* < 0.05) from exclusive nodal reads, and significant AS were mapped to the GO term (http://www.geneontology.org/). The significance of DSGs in KEGG pathways for genome KEGG background was tested (*p* < 0.05).

### 2.6 Genes of encoding salt stress regulators express patterns

To enable qRT-PCR analysis, cDNA synthesis was performed using total RNA extracted from *G. uralensis* as the template. This specific method is consistent with Li et al. (2022). The primers used are lists in Supplementary Table S1.

## 3 Results

### 3.1 Overview of RNA-seq datasets sequencing quality under salt stress conditions

**FIGURE 1:**
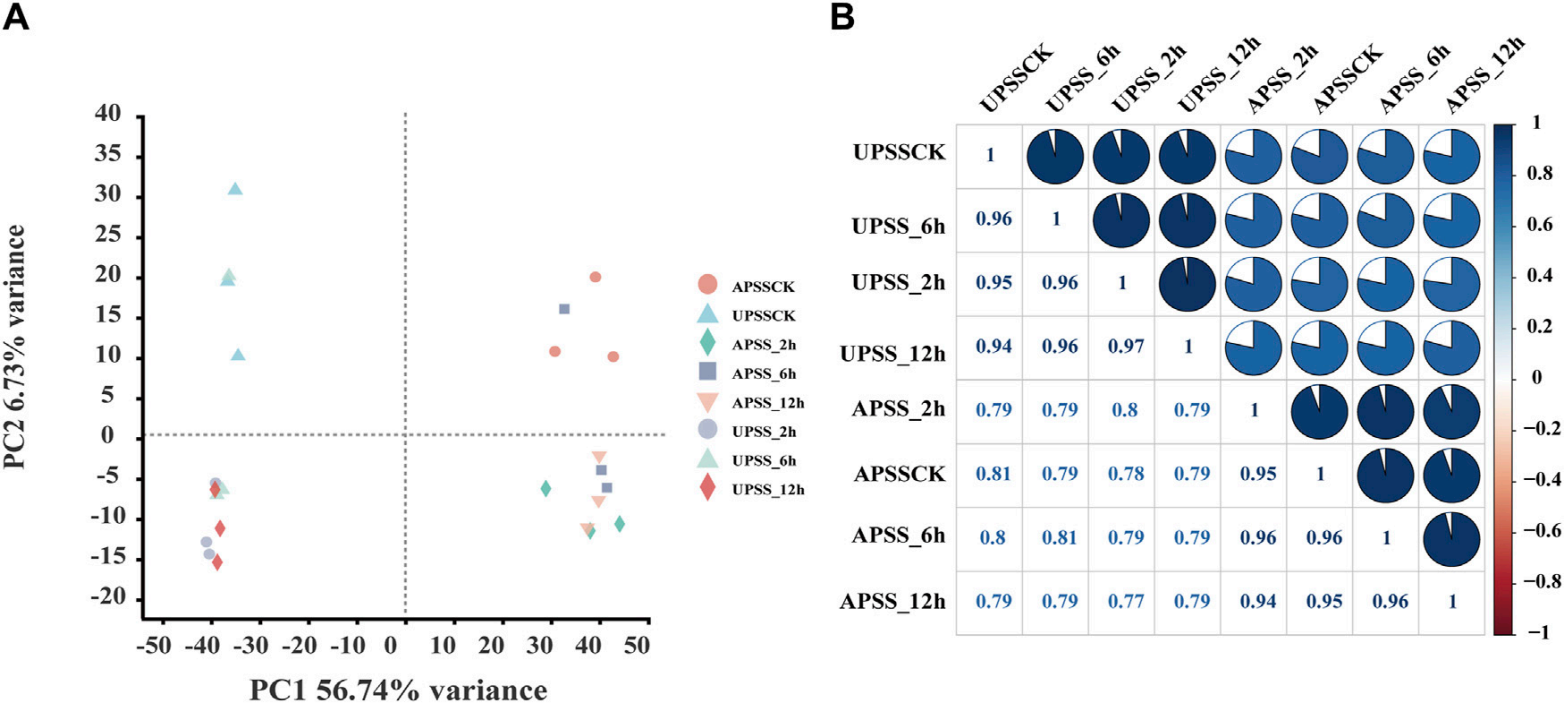
Assessment of RNA-seq dataset for identifying AS events. **(A)** Principal component analysis (PCA) of RNA-seq data. The sample replicates of the same group formed the same clusters and of different groups were classified into diverse clusters. **(B)** To assess the relationship between the samples, a correlation analysis of the RNA-seq was performed. Correlation matrices were generated by calculating the mean values of AP and UP at different time points under salt-treatment. Pearson correlation coefficients were computed using R scripts.

We analyzed AS events in *G. uralensis* in response to salt stress by utilizing 24 high-throughput RNA-seq libraries. The seedlings were exposed to salt stress at different time points: 0 (APSSCK and UPDSCK), 2 (APSS_2h and UPSS_2h), 6 (APSS_6h and UPSS_6h), and 12 h (APSS_12h and UPSS_12h). The analysis was performed using TopHat2 software, which has been proven superior to previously used TopHat software for analyzing soybean and *G. uralensis* RNA-seq data (Li et al., 2020; Song et al., 2020). We analyzed each sample using over 4.2 million clean reads. The presence of high percentages of Q20 (>98%) and Q30 (>94%) confirmed high sequencing accuracy (Supplementary Table S2), enabling further data analyses. The distribution of filtered reads across the first 20 chromosomes of *G. uralensis* was indicated (Supplementary Figure S1A). There was no conspicuous preference in the sequence coverage (Supplementary Figure S1B), allowing us to perform further data analysis for AS event identification. Principal component analysis (PCA) was performed to validate sample repeatability (Figure 1A). Clusters of biological replicates were observed to have close distances, indicating acceptable variation between different time points. In addition, the biological replicates showed strong correlations with correlation coefficients (r^2) ranging from 0.77 to 1 in AP and 0.94 to 1 in UP (Figure 1B). This indicated that these RNA-seq datasets were high-quality and reliable.

### 3.2 Identification of AS events in *G. uralensis* tissues under salt treatment

**FIGURE 2:**
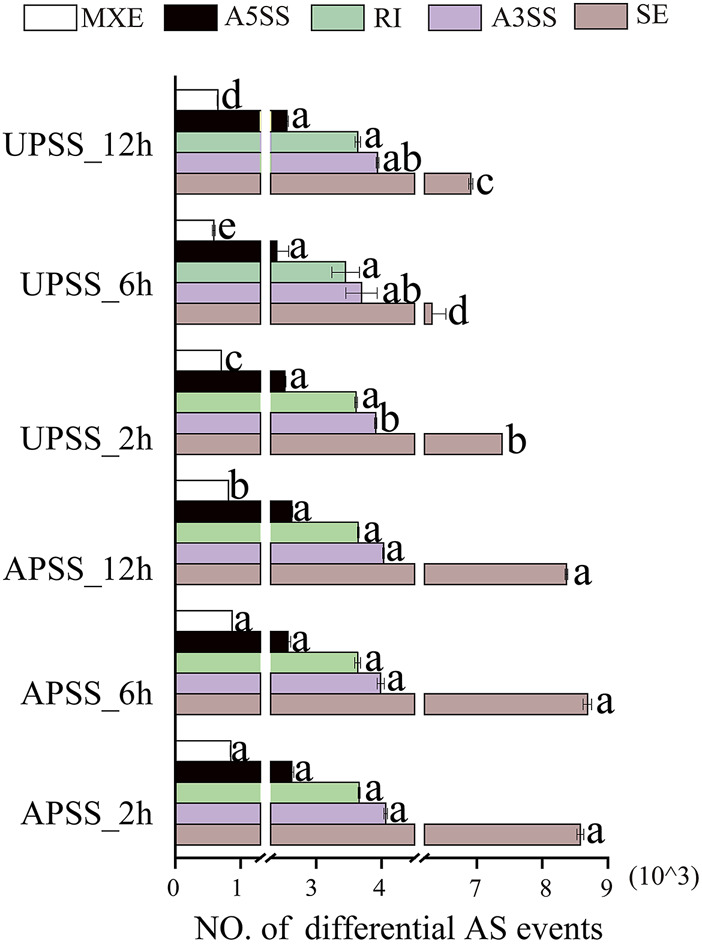
Quantities of various AS event types in salt-treated transcriptomes in *Glycyrrhiza uralensis*. Five AS event types of SE, MXE, A5SS, A3SS, and RI were analyzed, with the data of three biological replicates to be indicated as mean ± standard deviation. The mean values were evaluated by Bonferroni test, with distinct letters to represent significant differences among diverse treatments (*p <* 0.05).

We used 24 RNA-Seq datasets to analyze the comprehensive landscape of AS events in salt-treated *G*. *uralensis*. After mapping the high-quality reads to the reference genome of *G. uralensis* (Mochida et al., 2017), we utilized the rMATS software with the junction count-only setting to identify and quantify AS events, and tested the quantity of the five AS types (SE, MXE, A5SS, A3SS, and RI) using the Kolmogorov–Smirnov method (Supplementary Table S3). After correction using one-way ANOVA, the Bonferroni method was applied to the mean values (Supplementary Table S4). The results showed that salt treatment for 2, 6, and 12 h significantly increased the amount of SE, A3SS, A5SS, and RI AS events in the AP compared to the UP of *G. uralensis*. In the UP of salt-treated *G*. *uralensis*, significant differences (*p < 0.05*) in SE and MXE AS events were detected without significant differences in A3SS, A5SS, and RI AS events (Figure 2). When comparing the mean differences of five AS events in AP and UP, significant differences were identified in the mean discrepancies among the five AS events, especially in the UP of *G. uralensis*. Furthermore, significant mean difference in SE and MXE but not in RI, A3SS, and A5SS AS events were observed in the UP of salt-treated *G*. *uralensis* (Supplementary Figure S2). These results suggest that AS events are common during salt stress, with the UP being the major tissue site in *G. uralensis* for the response to salt.

### 3.3 Recognition of AS events responding to salt-treated *Glycyrrhiza uralensis* AP and UP sections

**FIGURE 3:**
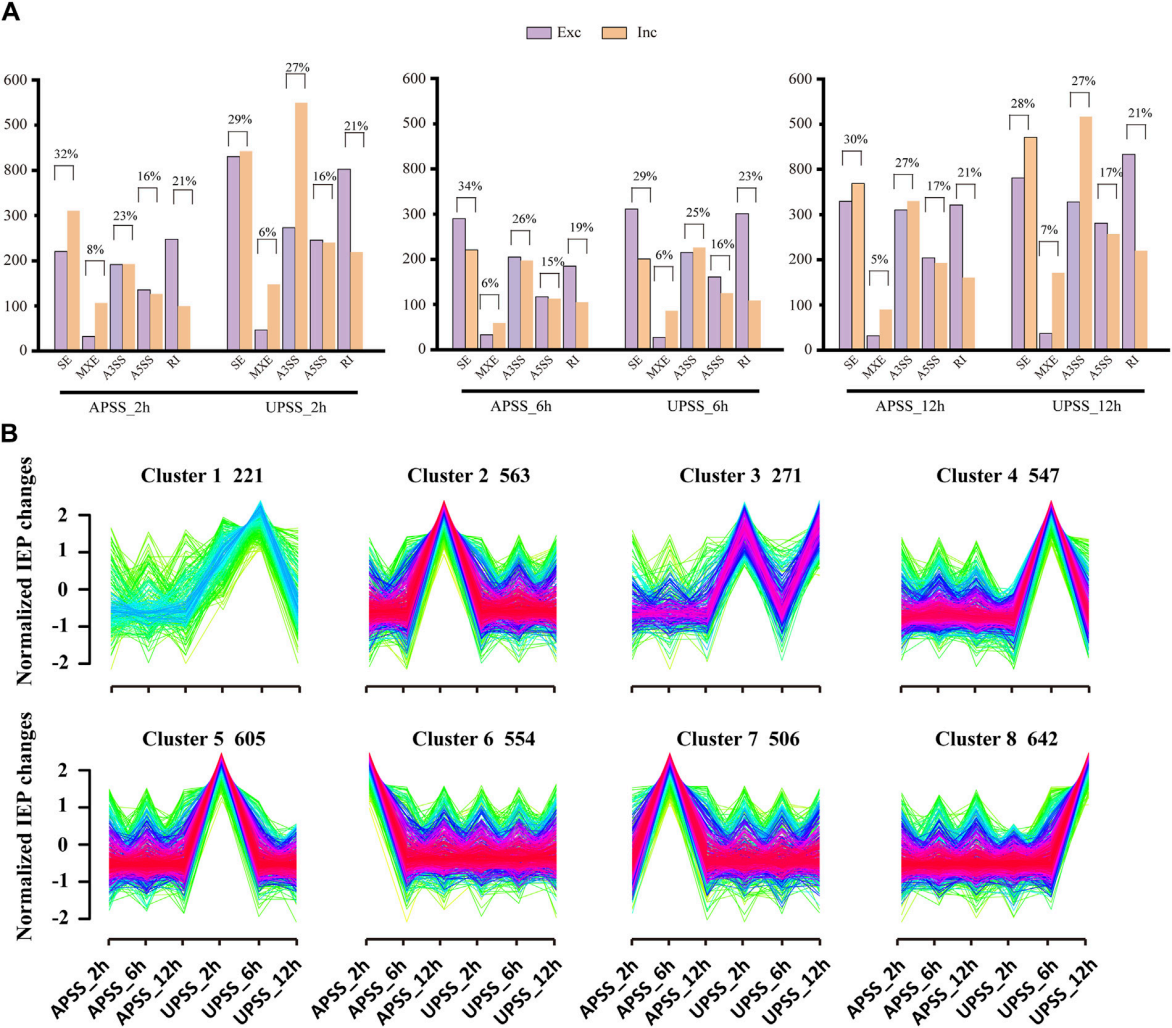
Identification and comparative analysis of the AS events in *G. uralensis* under salt stress. **(A)** Comparative analysis of the quantity of AS events in salt-treated *G. uralensis* UP and AP sections. **(B)** IEP-based cluster examination of AS events in *G. uralensis* in response to salt stress. The count of AS events in each cluster was indicated, with *x*-axis representing the different treatment time points in *G. uralensis* UP and AP sections and *y*-axis representing the IEP values. The red lines indicate the trend of IEP mean value for each AS event in different clusters.

Reports indicated that AS events in wheat roots led to diverse spliced gene isoforms under salt stress. Under drought stress, the generation of variant splicing isoforms in *G. uralensis* genes is similar Li et al. (2022). It suggest that these events contribute to *G. uralensis*’ responsiveness to salt. In this study, a total of 1648, 1505, and 2329 AS events in AP and 2983, 1742, and 3086 AS events in UP were obtained in salt-treated *G*. *uralensis* for different time points of 2, 6, and 12 h, respectively (Figure 3A). SE was the most prevalent AS event, accounting for 34% of AP and 29% of UP. Furthermore, following 2 h and 12 h of salt stress exposure in *G. uralensis* of AP tissue, the SE events significantly tend towards the inclusion type. In contrast, the same events in UP leaned towards the exclusion type (Figure 3A). Hence, the abundance of diverse isoforms varies widely across different anatomical regions of *G. uralensis* plants.

Isoform expression percentage (IEP) alterations have been used to categorize AS events in wheat (Li et al., 2022) and soybean (Song et al., 2020) under salt stress. In *G. uralensis* AP and UP sections, SE-type AS events emerged as the primary manifestation of salt-related AS in response to salt stress. Consequently, we utilized Mfuzz software to perform the IEP cluster analysis in SE-type with the conditions of “*FDR* < 0.05” and “change in isoform expression percentage ≥30%” (Figure 3B; Supplementary Table S5). The results indicated that eight groups were classified representing 221, 563, 271, 547, 605, 554, 506, and 642 AS events, respectively. Group 3 exemplified changes in the AS pattern at the UPSS_2 h, UPSS_6 h, and UPSS_12h time points. Similarly, groups 1, 4, 5, and 8 showed variations in AS patterns in the salt-stressed *G. uralensis* UP section, revealing their sensitivity to salt stress. Conversely, Groups 2, 6, and 7 exhibited minimal adjustments in the AS pattern in the salt-stressed AP tissues of *G. uralensis*. Therefore, significant alterations in AS patterns in response to salt stress occurred mainly in *G. uralensis* UP tissues. These results demonstrated that the substantial AS pattern alterations in response to salt stress occurred predominantly in *G*. *uralensis* UP.

### 3.4 Comparative evaluation of differentially expressed and spliced genes

**FIGURE 4:**
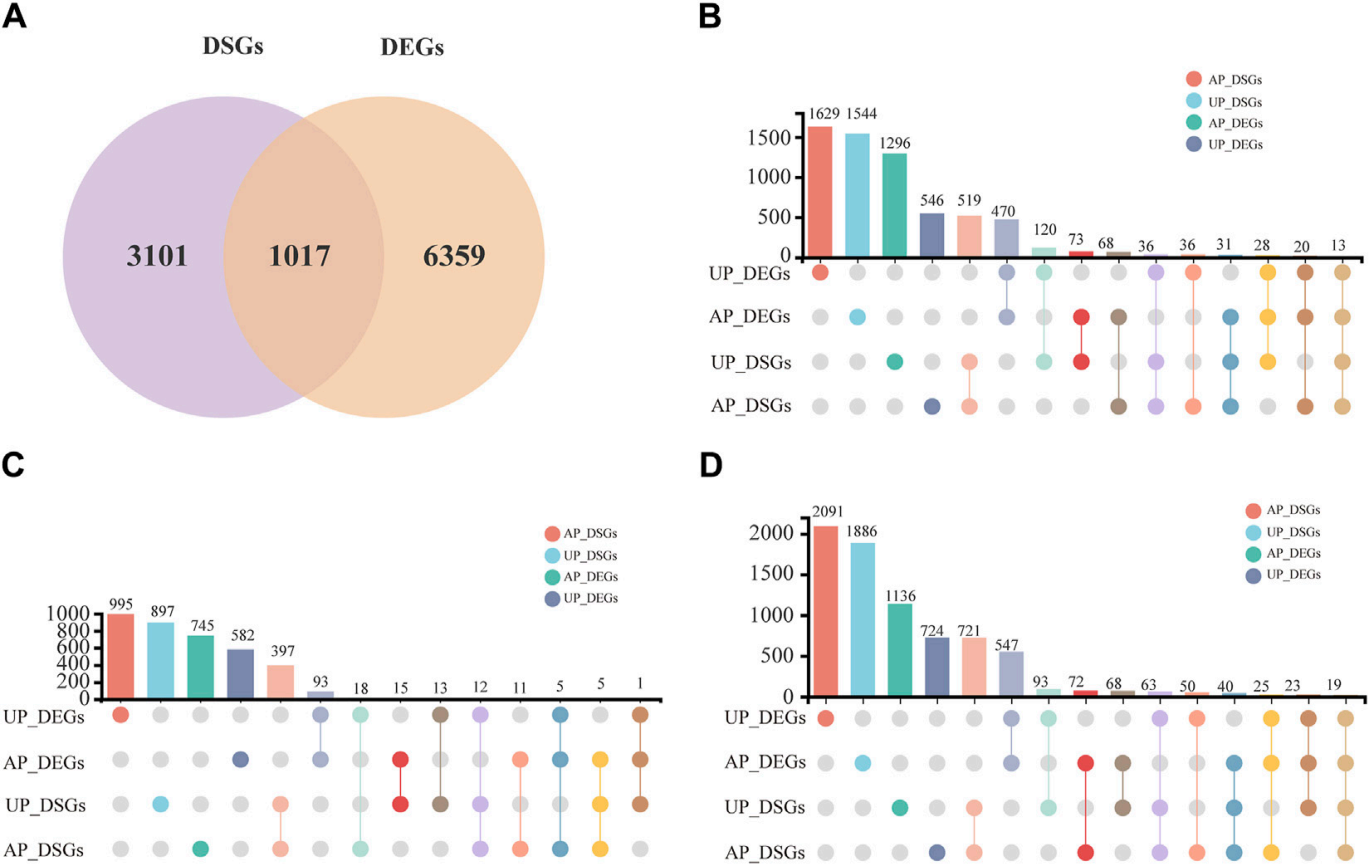
Analysis of DSGs and DEGs in salt-treated *G. uralensis* AP and UP sections. **(A)** Comparative analysis was performed on the overlapping DEGs and DSGs subjected to different salt stress treatments. The DSGs and DEGs in the *G. uralensis* AP and UP sections were measured at 2 h **(B)**, 6 h **(C)**, and 12 h **(D)** intervals during salt treatment.

DSGs were generated due to AS events and exhibited significant variation under salt stress. Overall, 4,117 DSGs in *G. uralensis* were identified and distributed across various phases throughout the salt stress (Supplementary Table S6). Of these, 1,017 genes showed clear salt-responsive and AS regulatory expression patterns (Figure 4A). In order to better understand the AS events in response to salt treatment, we compared the levels of DSGs and DEGs in salt-stressed *G. uralensis* UP and AP tissues at various time intervals throughout the salt stress treatment period (Figures 4B–D; Supplementary Table S6). We observed 68, 11, and 72 genes evident in DSGs and DEGs in AP tissues at 2, 6, 12 h. Similarly, 120, 13, and 93 genes were identified in UP tissues as DSGs and DEGs at 2, 6, and 12 h, respectively (Figure 4B). These results showed that salt-responsive expression tendencies and AS regulation were more significant in 2-h and 12-h treated tissues. Furthermore, the newly discovered AS events were analyzed in *G*. *uralensis* under salt stress; the results indicated that 1,746, 356, and 150 novel AS events of the SE, MXE, and RI types were identified and had a significant response (Supplementary Table S7), highlighting the substantial involvement of SE-type AS in *G. uralensis* salt stress response.

### 3.5 GO analysis of DSGs in salt-treated *G. uralensis* AP and UP

**FIGURE 5:**
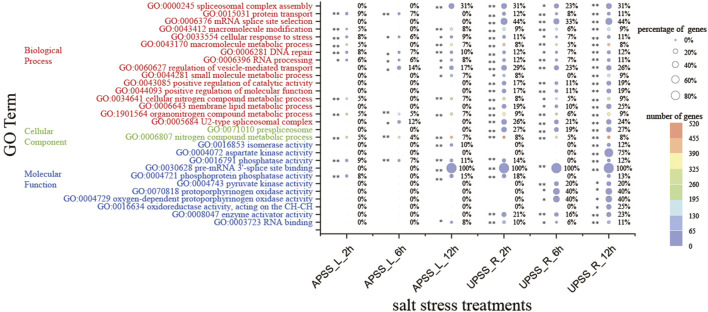
GO analyses of DSGs under salt stress. **(A)** GO enrichment analysis of DSGs. DSGs were subject to GO terms with the condition of FDR < 0.05. **(B)** Top 20 GO terms of the DSGs in the category of molecular function. **denotes *p* < 0.01 and * represents *p* < 0.05*.*

In order to comprehend the impact of AS regulation on the biological functions of *G. uralensis* during the process of salt stress response, a GO enrichment analysis was performed on all DSGs. The results showed that numerous DSGs regulated by AS were related to metabolic, spliceosome, and oxidase activity (Figure 5). A total of 19 GO terms were significantly enriched in underground tissues, including cellular response to stress, DNA repair, and organonitrogen compound metabolism. The AP tissues exhibited significant enrichment of six GO terms, including metabolic processes for nitrogen compounds, phosphatase activity, and RNA processing. GO term enrichment in UP tissues is much higher than in AP tissues. Salt stress-related GO terms were only significantly enriched in UP tissues, not AP tissues, such as protoporphyrinogen oxidase activity, oxygen-dependent protoporphyrinogen oxidase activity, and oxidoreductase activity. It is possible that UP tissues first experience salt stress and then send signals to ground tissues. Furthermore, DEGs from UP and AP tissues in salt stress conditions were analyzed using GO to assess their functional significance. There was a significant enrichment in GO terms related to salt stress, such as oxidoreductase activity, flavonoid glucuronidation, and oxidoreductase activity, acting on CH–OH (Supplementary Figure S3). The enriched regulation of intracellular metabolic, spliceosome, and oxidase activity provides a comprehensive understanding of how AS regulates the salt stress response in *G. uralensis*.

### 3.6 KEGG enrichment analysis of DEGs and DSGs that encode splicing regulators

**FIGURE 6:**
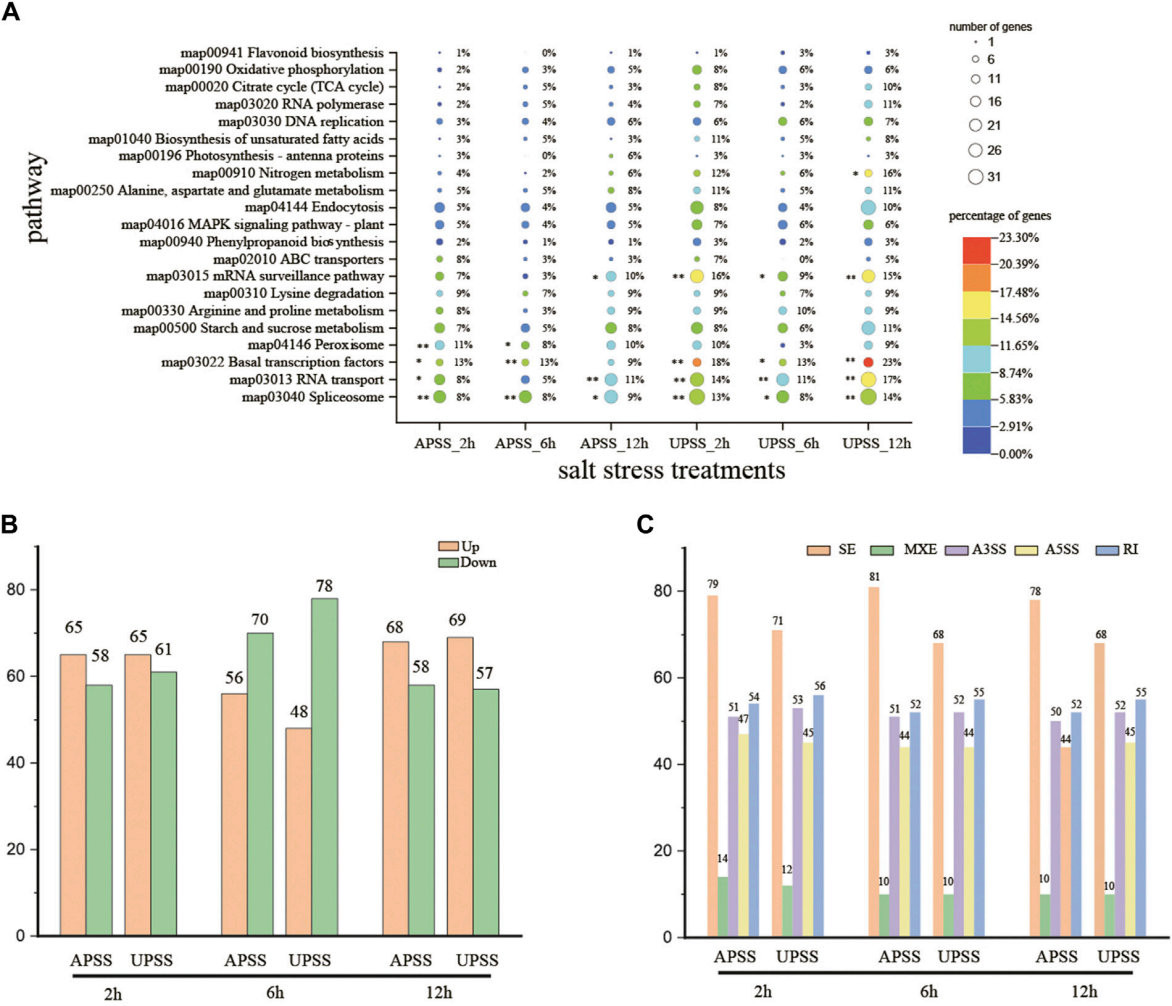
Analysis of KEGG enrichment and AS pattern of SPF-related genes during salt stress. **(A)** Top 20 pathways demonstrated enrichment of DSGs. **(B)** Notable differential splicing was observed within SPF-related genes under salt-stress conditions. **(C)** Significant differential expression was recorded within SPF-related genes under salt stress. **denotes *p* < 0.01 and * represents *p* < 0.05.

Under various stress conditions, salt stress triggers the response of splicing regulatory factors (SPFs) and induces diverse patterns of splicing (Ding et al., 2014; Filichkin et al., 2010; Li et al., 2022; Palusa et al., 2007; Tanabe et al., 2007). In our work, KEGG enrichment analysis was performed on the difference splicing genes (DSGs). Pathways associated with AS, such as RNA transport, mRNA surveillance, and spliceosome, were significantly enriched, indicated splicing regulation of genes and resulting in AS events under salt stress conditions. These results indicated that the SPFs-encoding genes appeared to respond to salt stress in *G*. *uralensis* UP sections and exhibited variable splicing (Figure 6A). Moreover, plant response to salt stress pathways were also enriched, such as MAPK signaling pathway-plant, oxidative phosphorylation, and flavonoid biosynthesis. A total of 81 SPF-related genes and 45 genes related to salt stress that displayed significant splicing variation at diverse salt treatment time points were discovered (Supplementary Table S8). After salt stress treatment for 2 and 12 h, there were more upregulated than downregulated genes (Figure 6B). The main AS events were SE and RI in salt-treated *G*. *uralensis* (Figure 6C). Most SPF-related genes showed increased expressions in salt-treated *G*. *uralensis* UP section (UPSS_2h, UPSS_6h, and UPSS_12h) (Supplementary Figure S4). These results suggest that the SPF-encoding genes and genes related to salt stress are regulated by AS in *G*. *uralensis* UP under salt stress.

To comprehend how stress response genes in the root tissue of *G. uralensis* adapt to salt stress, we examined the expression levels of four genes encoding SPFs. As illustrated in Supplementary Figure S5, under salt stress at various time points, the four genes—*Glyur000002s00000283* (*similar to Glycine rich RNA-binding protein RZ1B*), *Glyur000834s00025309* (*akin to Polyglutamine-binding protein 1*), *Glyur000659s00029682* (*paralleling Serine/threonine protein phosphatase PP2A*), and *Glyur000136s00007936* (*reminding of Polyadenylate binding protein 2*)—exhibited high expressions as isoform 1 (spliced partial protein) and low expressions as isoform 2 (non-spliced intact protein) (Supplementary Figures 5A–D). These SPF-encoding genes displayed comparable responses to salt stress, implying their potential functions in modulating the salt-stress response in *G. uralensis*.

### 3.7 Identification of potential regulators in *G. uralensis* in response to salt stress

Many genes encoding the calcium ion and MAPK signal transduction pathway proteins showed notable AS alterations (Supplementary Figure S6). Glyur001147s00029003 (*MPK4*) encodes mitogen-activated protein kinase, with a significantly higher proportion of isoform 1 observed under salt stress 2, 6, and 12 h treatment. Under APSS_2h, APSS_12h, UPSS_2h, UPSS_6h, and UPSS_12h treatment conditions, there was a significant increase in isoform 1 of *Glyur000143s00011203 (RbohD). Glyur000099s00014961*(*SnRK2*) encodes erine/threonine–protein kinase, in which isoform 1 was detected in higher proportions under APSS_2h treatment conditions than control conditions. APSS_2h UPSS_2h, UPSS_6h, and UPSS_12h treatment conditions resulted in significantly higher proportions of isoform 1 than control conditions for *Glyur000837s00027495* (*ETR2*) (Supplementary Figures S6C,E,F,G).

**FIGURE 7:**
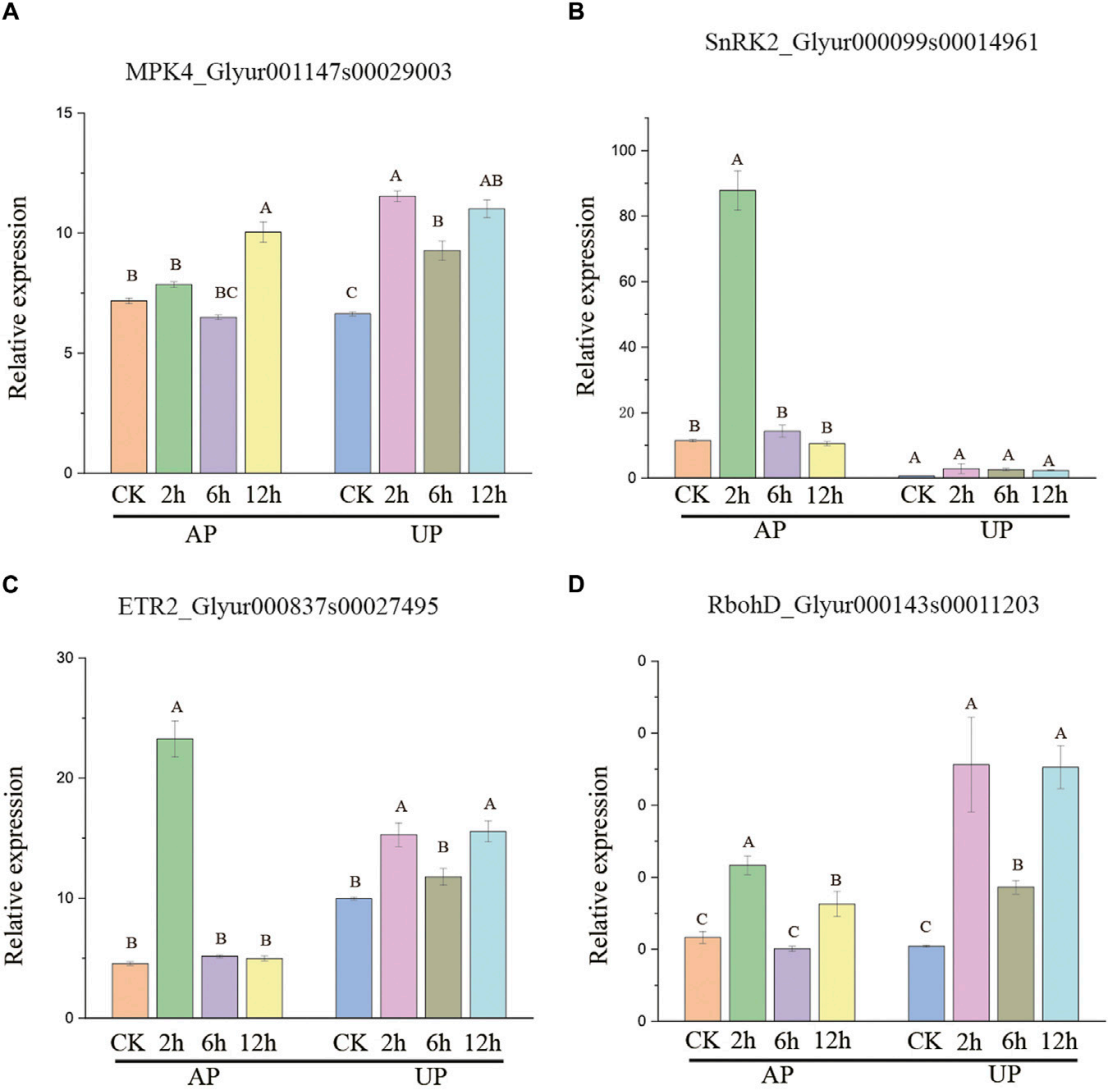
Expression levels of *BAM1* **(A),** cyclin-dependent kinase B2-2 (*CDKB2-2*) **(B)**, *DRM2* **(C)**, and *PP2C12* **(D)** in salt-stressed *G. uralensis* AP and UP sections. Expression value was mean ± SD with three independent replicates. Bonferroni test used to analyze average value. Diverse letters denote significantly different salt stress treatments (*p* < 0.01).

The expression patterns of the four genes in the above-mentioned MAPK pathway were also examined using qRT-PCR under salt stress. *MPK4* had a significantly higher expression level observed under salt stress 2, 6 and 12 h treatment than control conditions. Under APSS_2h, APSS_12h, UPSS_2h, UPSS_6h, and UPSS_12h treatment conditions , there was a significant increase in expression level of *RbohD* compared to control conditions. *SnRK2* expression level was higher under APSS_2h treatment conditions than control. APSS_2h, UPSS_2h, UPSS_6h, and UPSS_12h treatment conditions resulted in significantly higher proportions of expression level than control conditions for *ETR2* (Figure 7). *G*. *uralensis* may be able to respond better to salt stress by increasing the proportion of isoform 1 of salt-stress-related genes. These findings indicate that significant alternative splicing events in the *MPK4*, *RbohD*, *SnRK2*, and *ETR2* genes are especially generated in *G*. *uralensis*, which lead to increased protein isoform 1 production in response to salt stress.

## 4 Discussion

### 4.1 Salt stress significantly induced AS events in *G. uralensis*

AS events are an important factor for plants to respond to stress; RNA-seq studies-based analysis of abiotic stress-regulated AS events in *G. uralensis* is still limited (Calixto et al., 2018; Ding et al., 2014; Li et al., 2020; Liu et al., 2018; Thatcher et al., 2016). A total of 2591 and 3068 AS events were identified in the aboveground parts (AP) and underground parts (UP) of *G*. *uralensis* under salt stress, respectively, including SE, RI, A5SS, A3SS, and MXE. Under salt stress, the UP exhibited a higher occurrence of AS events related to stress response in comparison to the AP, indicating a more vigorous AS response. Some AS events may be gene-specific and some species-specific, with SE and RI being the most and least prevalent respectively in animals. The majority of AS cases are caused by SE, while only 0.01% are caused by RI; in contrast, RI is the most common form of AS in *Arabidopsis* and maize (Thatcher et al., 2016). In recent years, however, SE and RI have been the most prevalent AS in licorice and soybeans (Li et al., 2022; Song et al., 2020) but MXE the least. These results are in line with our study. This may be due to advances in sequencing technology and detection tools that allow for more detailed detection of AS. Among these, the SE-types of AS were the most prevalent. In AP tissue, SE-types accounted for 30%–34% of the responsive AS events, while in UP tissue they accounted for 28%–29% of the responsive AS occurrences in salt-stressed *G. uralensis*. Under salt stress, there was a higher occurrence of A3SS events than RI events (Figure 2), but the count of A3SS and RI events was not significantly different (Figure 2). This finding aligns with a previous study that reported SE and A3SS as the predominant AS models in soybeans and *G. uralensis* under drought stress. However, RI was the notable AS type in reaction to drought in soybeans. Under high temperatures and drought treatments in wheat, the predominant type of AS was found to be RI, whereas the primary model in maize of AS in coping with stress was also RI (Iniguez et al., 2017; Liu et al., 2018; Shen et al., 2014; L; Wang et al., 2014; Y; Wang et al., 2020). These differences in the major AS types could be attributed to variations in SRP-related genes. Consequently, our investigation revealed many AS events in *G. uralensis* under salt stress condition, underscoring their crucial contribution to the response of *G. uralensis* to saline environment pressures.

### 4.2 DSGs enriched in key GO terms and KEGG pathways associated with salt stress and AS

Global crop production is severely constrained by salt stress, which negatively impacts the growth of *G. uralensis* (Xiao et al., 2024). There are many genes that are responsive to salt stress, but the alternative splicing patterns of these are still unclear. In this study, we found that DEGs and DSGs mainly occurred in the root (Figures 4B–D). To investigate what functions these DEGs and DSGs have, we analyzed their GO enrichment separately. The results indicate that most of these GO terms are enriched in UP tissues. In DSGs, GO terms such as “pyruvate kinase activity”, “protoporphyrinogen oxidase activity”, and “protoporphyrinogen oxidase activity” were significantly enriched in UPSS_6h and UPSS_12h (Figure 5). In DEG, GO terms such as “isoflavonoid biosynthetic process”, ”flavonoid glucuronidation”, “oxidation-reduction process”, and “isoflavonoid metabolic process”, consistent with previous studies, all participated in salt stress response (Kalifa et al., 2004; Kononenko et al., 2023; Tavakkoli et al., 2011; Zhang et al., 2023). Meanwhile, in DSGs, some GO terms such as “spliceosomal complex assembly”,“U2-type spliceosomal complex”, “prespliceosome”, and “pre-mRNA 3′-splice site binding” were significantly enriched at UPSS_2h, UPSS_6h, and UPSS_12h especially, and “mRNA splice site selection” was enriched at UPSS_2h, UPSS_6h, and UPSS_12h in *G. uralensis* (Figure 5), which was known to be important for alternative splicing (Clayton et al., 2011; Schindler et al., 2008). Interestingly, these GO terms were not enriched in the DEG. These results indicate that the alternative splicing genes played important roles in the response to salt stress. Moreover, some previously identified stress-related proteins were also enriched, such as enzyme activator activity (Gao et al., 2017).

To further investigate on which signaling pathways these DSGs function, we performed KEGG enrichment. We observed remarkable enrichment of DSGs in the “RNA transport”, “mRNA surveillance”, and “spliceosome” pathways, which were significantly enriched in UPSS_2h, UPSS_6h, and UPSS_12h, which are known to be important for alternative splicing (Liu et al., 2018; Song et al., 2020). The regulation of alternative splicing represents an important means of fine-tuning gene expression that may save the time required for changes in transcriptional activation and pre-mRNA accumulation, thus allowing rapid plant adaptation to adverse environmental conditions. Ultimately, the effects of alternative splicing on mRNAs’ encoding effectors and modulators of abiotic stress responses are determined by the levels and/or activity of the splicing factors regulating this process. Pre-RNA undergoes maturation to become mature mRNA through the spliceosome process, making it an important splicing process (Syed et al., 2012). Marondedze et al. (2019) recently reported that spliceosome component modifications were consistent with changes in drought-stressed *Arabidopsis* proteome and transcriptional profiles. We also discovered that genes encoding SPFs proteins, which these are certain RNA-binding (*Glyur000002s00000283*, *Glyur000834s00025309*, and *Glyur000136s00007936*) were potentially regulated by AS. Spliceosomes have been reported in *G. uralensis* under drought stress (Li et al., 2022), indicating their involvement in the reaction of *G. uralensis* to salt stress.

The mRNA surveillance pathway detects and degrades abnormal mRNAs and plays an important role in maintaining accurate gene expression with salt stress in soybeans (Song et al., 2020). In soybeans, nonsense-mediated decay (NMD) is a crucial pathway responsible for mRNA surveillance and facilitates the degradation of mRNAs that contain premature termination codons (Lareau et al., 2007). According to Baena-Gonzalez et al. (2007), approximately 17.4% of multi-exonic and protein-coding genes in *Arabidopsis* are known to produce splicing variants aimed by NMD. Salt stress modulates AS in conjunction with NMD in *Arabidopsis* (Drechsel et al., 2013). In the present study, we identified one gene (*Glyur000659s00029682*) that encodes the serine/threonine PP2A regulated by salt stress and AS (Supplementary Figure S4D). Hence, the salt stress response in *G. uralensis* could potentially be regulated by the mRNA surveillance pathway. Pre-mRNAs and proteins abundant in Ser/Arg residues are key players in AS, helping maintain cellular and tissue homeostasis (Ding et al., 2014; Laloum et al., 2018; Li et al., 2022; Reddy et al., 2011). AS occurs in SRP-related genes in plants in a developmental and tissue-specific manner, responding to various hormonal and abiotic stresses (Ding et al., 2014; Li et al., 2022; Zhang et al., 2009; Zhu et al., 2017). BrSR45a was found by Muthusamy et al. (2020) to increase stress-inducible genes and influence the AS of target genes in *Arabidopsis*. Interestingly, our study discovered that AS during salt stress affected specific genes involved in encoding Ser/Arg-rich proteins, which are identified as participants in pre-mRNA splicing (Figure 6). Therefore, SRP-related genes in *G. uralensis* may play a significant role in coping with salt stress.

Some KEEG pathways such as “Peroxisome” were significantly enriched at APSS_2 h and APSS_6h, which was consistent with previous studies that all participated in salt stress response (Liu et al., 2018). Moreover, some previously identified stress-related signaling pathways were also enriched, such as MAPK signaling pathway – plant (Yang et al., 2018). Plants have collectively evolved specific responses at both transcriptional and AS levels to cope with salt stress.

### 4.3 Signaling pathways in response to salt stress

In addition to AS, salt stress can also give rise to oxidative stress by increasing the levels of reactive oxygen species (ROS) (Carillo, 2019). Plant antioxidant systems have been shown to effectively reduce the effects of oxidative stress and reduce ROS formation (Farhangi-Abriz et al., 2017). Long-term evolution has led to a variety of adaptive physiological and biochemical strategies for plants, including protecting against high-salt environments, restoring ROS equilibrium, and maintaining ion and osmotic homeostasis. Plants respond to salt stress by producing H_2_O_2_ from NADPH oxidase (Rboh) (Liu et al., 2020). *RbohD* under APSS 2,12, UPSS 2,6,12 elevated the proportion of isform 1 through A5SS events and then increased its expression. The excess H_2_O_2_ was then removed to improve salt stress response in plants.

Licorice increases antioxidant activity when exposed to salt stress conditions and reduces lipid peroxidation due to free radical damage (Xu et al., 2021). In response to salt stress, MAPK cascades are triggered, such as on the SOS pathway (Yang et al., 2018c). Plants can adapt to salt stress by utilizing MAPK cascades for transducing signals (Mishra et al., 2006). Salt stress is regulated by the *MPK4* cascade in *Arabidopsis*. Plants with the MPK4 gene null showed hypersensitivity to salt stress, whereas plants with overexpressed MPK4 showed greater salt tolerance (Teige et al., 2004). There are other plants that also rely on the MAPK cascade for salt stress signal transduction. Alfalfa (*M. sativa*), for example, uses the MKK-MK cascade to trigger its response to salt stress (Kiegerl et al., 2000). The *MPK4* cascade controls salt stress signal transduction in rice by regulating the expression of transcription factor genes (F. Wang et al., 2014). When *Arabidopsis* is dehydrated, mutants of the MKK4 cascade are more salt sensitive and shed more water than the wild type (Kim et al., 2012). We demonstrate that MPK 4 under salt stress increases isform 1 through SE events, increases its expression, and regulates downstream genes responding to salt stress to improve plant tolerance. When *Asterochloris erici* is subjected to hyperosmotic stresses such as high salt and desiccation, MAPK signaling cascades are activated (Gasulla et al., 2016). More studies are needed to better understand how salt/osmotic stress in plants affects MAPK signaling pathways. As a result of osmotic stress, all ten *SnRK2* isoforms are activated except *SnRK2*.*9*. The signaling pathway is dependent on ABA in order to activate *SnRK2.2/3/6/7/8* (Boudsocq et al., 2004; Nykiel et al., 2023). Osmotic stress activates transcription of downstream effectors through ABAactivated *SnRK2.2/3/6*-ABA-responsive element (ABRE)-binding protein and ABRE-binding factor (AREB/ABF) signaling (Soma et al., 2017). The moss *Physcomitrella patens* expresses an ABA-responsive Raf-like kinase (*ARK*) that activates *SnRK2* in response to osmotic stress (Saruhashi et al., 2015). As part of our research, we found that *SnRK2* enhanced isform 1 proportions by enhancing SE event expression, regulating downstream salt stress response genes, and improving salt stress tolerance in plants. Furthermore, many studies have shown that the ethylene (ET) synthesis genes *ETR1* and *ETR2* also regulate the synthesis of ABA and that ABA can interact with ET to regulate salt tolerance in plants (Zhang et al., 2022). *ETR2* at APSS _ 2 h, UPS SS _ 2, and 12h increased the proportion of isform 1 by RI events to improve ET synthesis and then regulate downstream salt stress response genes to improve plant tolerance to salt stress. In this study, expression levels of *MPK4*, *RbohD*, *SnRK2*, and *ETR2* were markedly elevated under CK treatment compared to salt stress, demonstrating a significant increase (Figure 7). In the root tissues of *G. uralensis*, these four genes exhibited significant AS events that produced additional proteins in response to salt stress. Furthermore, researchers can examine plant transcriptome datasets to analyze AS events and their variations across different species, tissues, and developmental stages. By examining AS patterns in various environments, researchers can uncover insights into the functions and mechanisms of AS in reaction to abiotic stress. Enhanced comprehension of this matter could potentially pave the way for the identification of novel strategies to bolster plant resistance against stress, thus advancing our knowledge of plant biology and augmenting our capacity to cultivate more resilient crops.

## Data Availability

The datasets presented in this study can be found in online repositories. The names of the repository/repositories and accession number(s) can be found in the article/Supplementary Material.
