# Supplementary figures and images for: Alternative splicing responses to salt stress in Glycyrrhiza uralensis revealed by global profiling of transcriptome RNA-seq datasets
Source: Front Genet. 2024 Jul 9;15:1397502. doi: 10.3389/fgene.2024.1397502 (PMC11263197; doi:10.3389/fgene.2024.1397502)

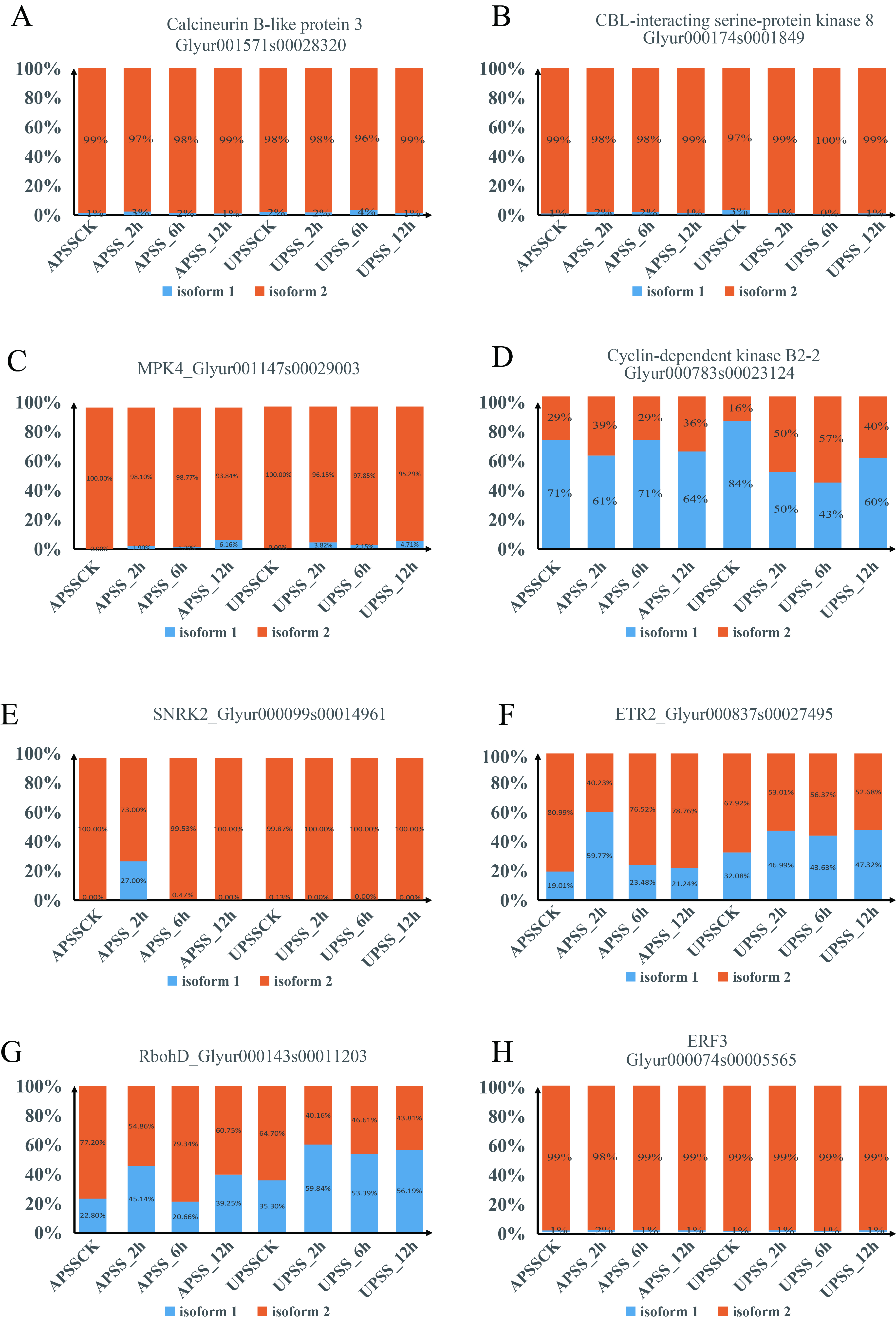

Supplement: Supplementary file 1 [file Image6.jpg]

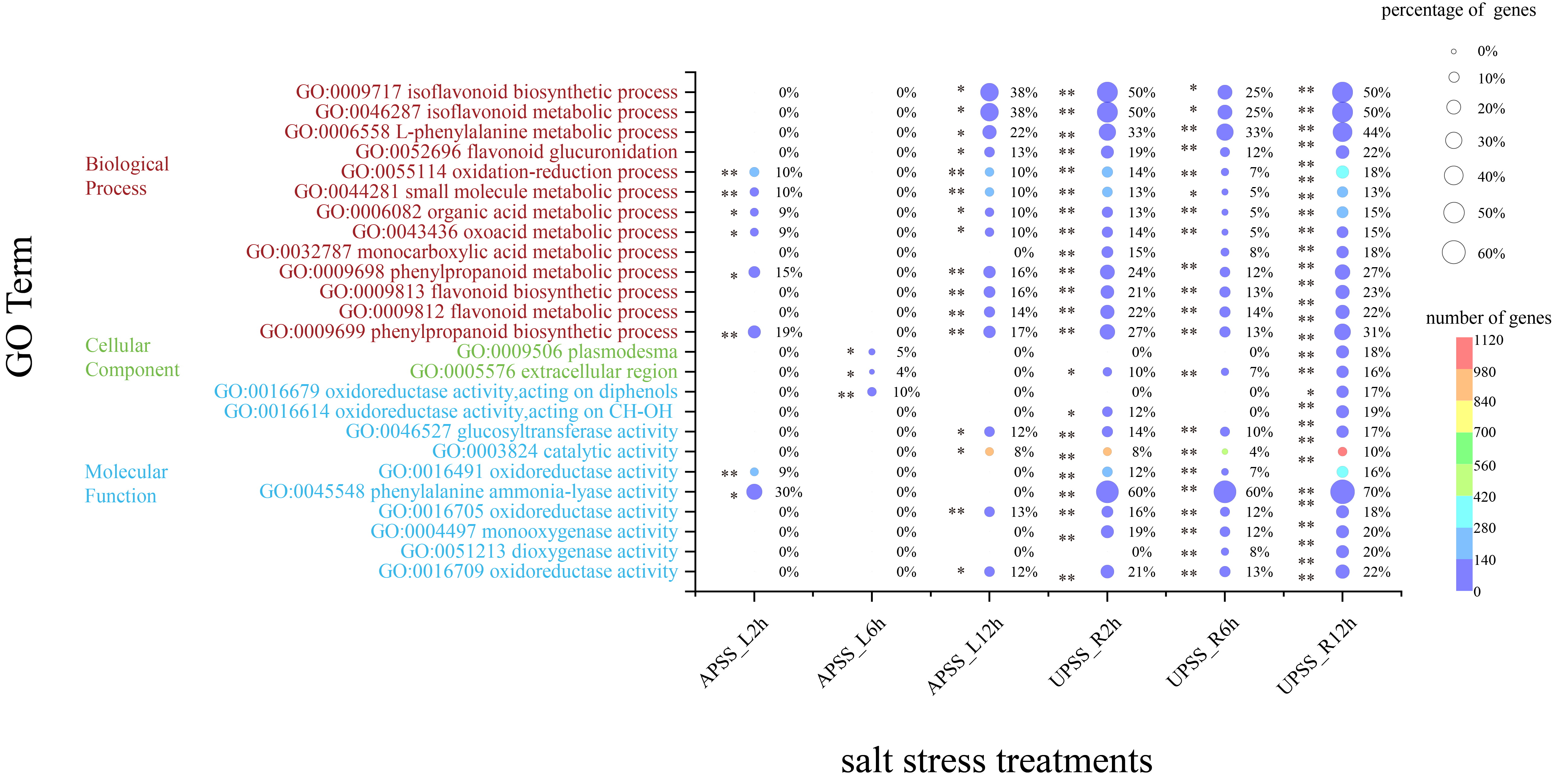

Supplement: Supplementary file 2 [file Image3.jpg]

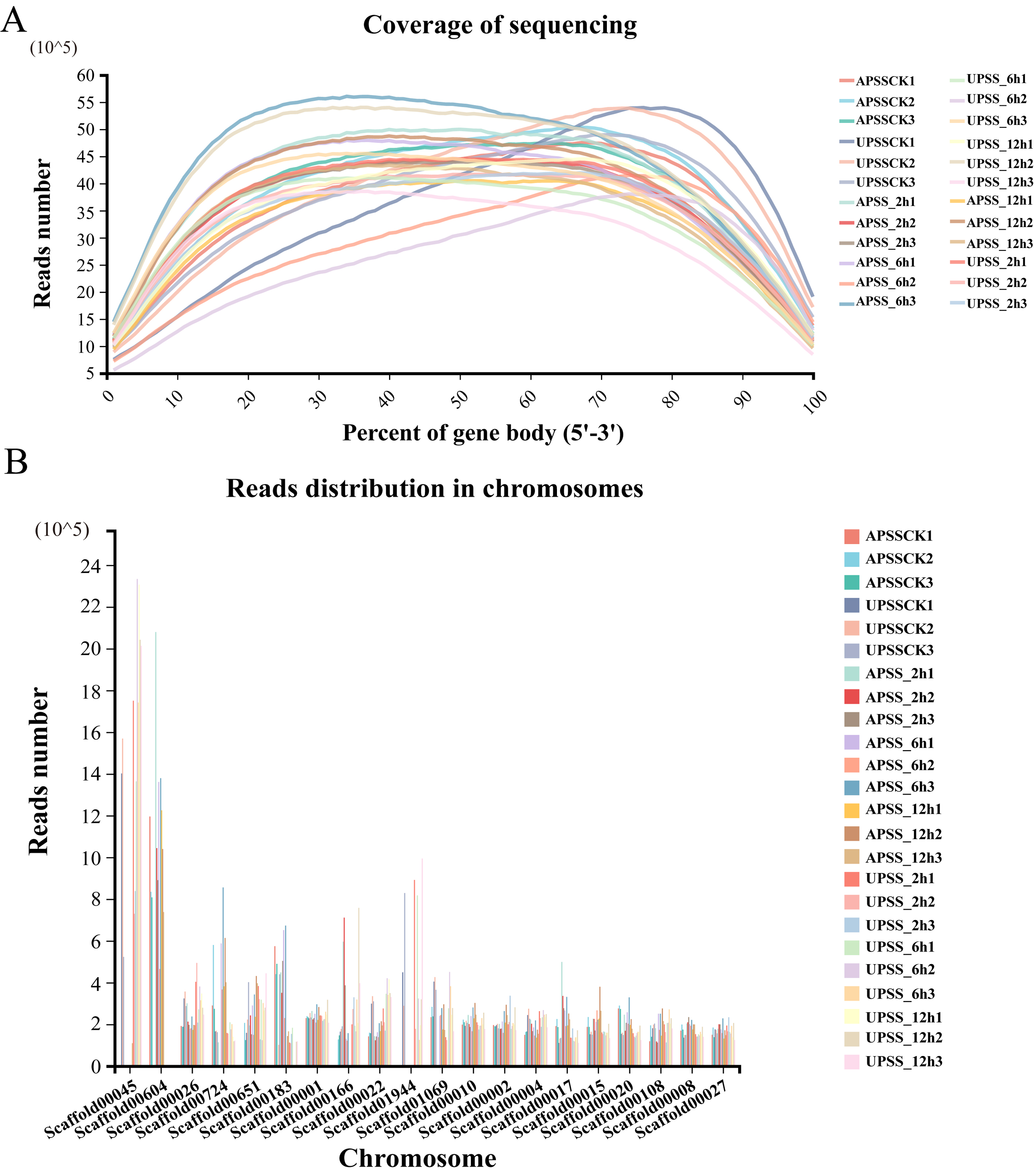

Supplement: Supplementary file 3 [file Image1.JPEG]

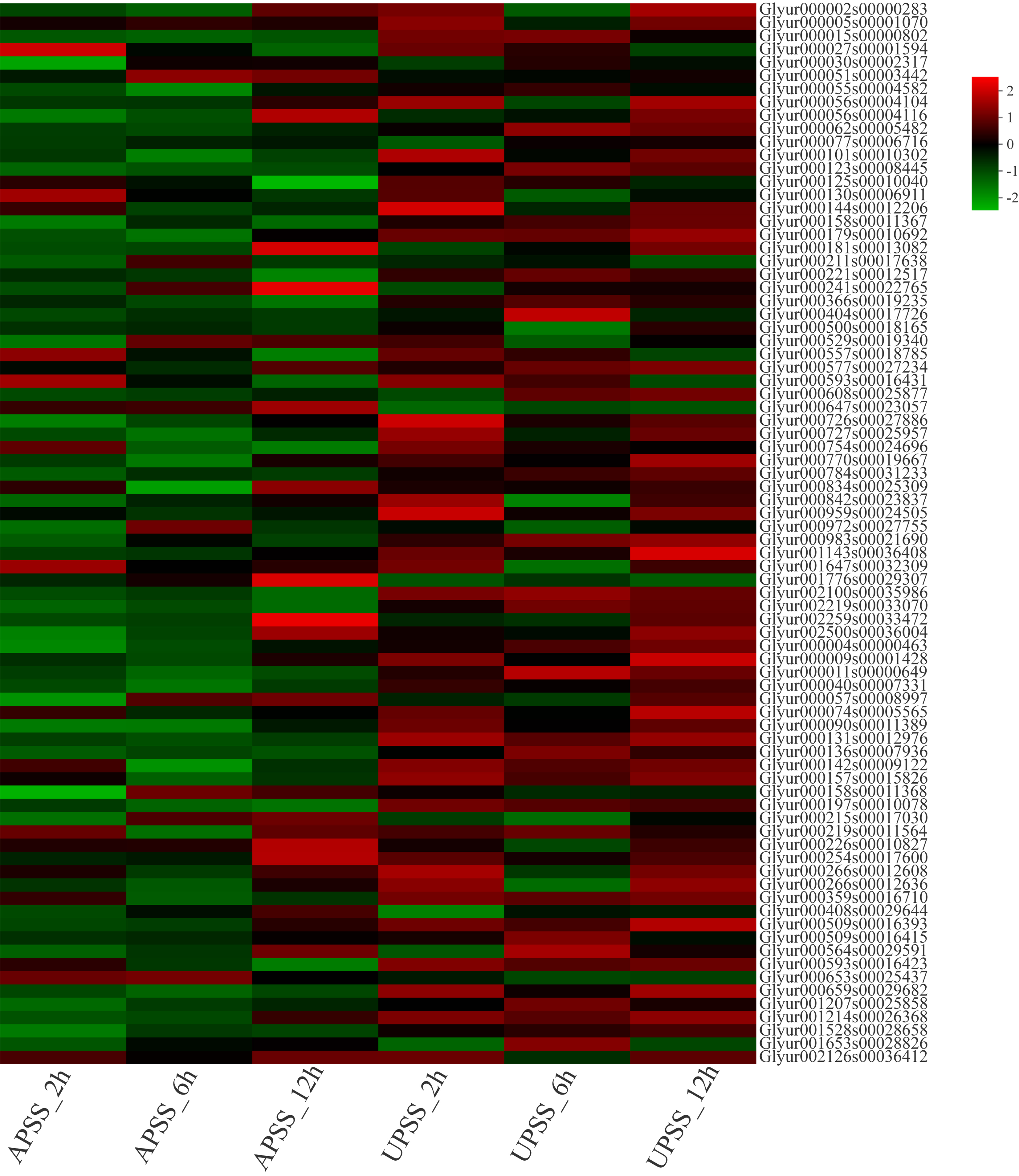

Supplement: Supplementary file 4 [file Image4.JPEG]

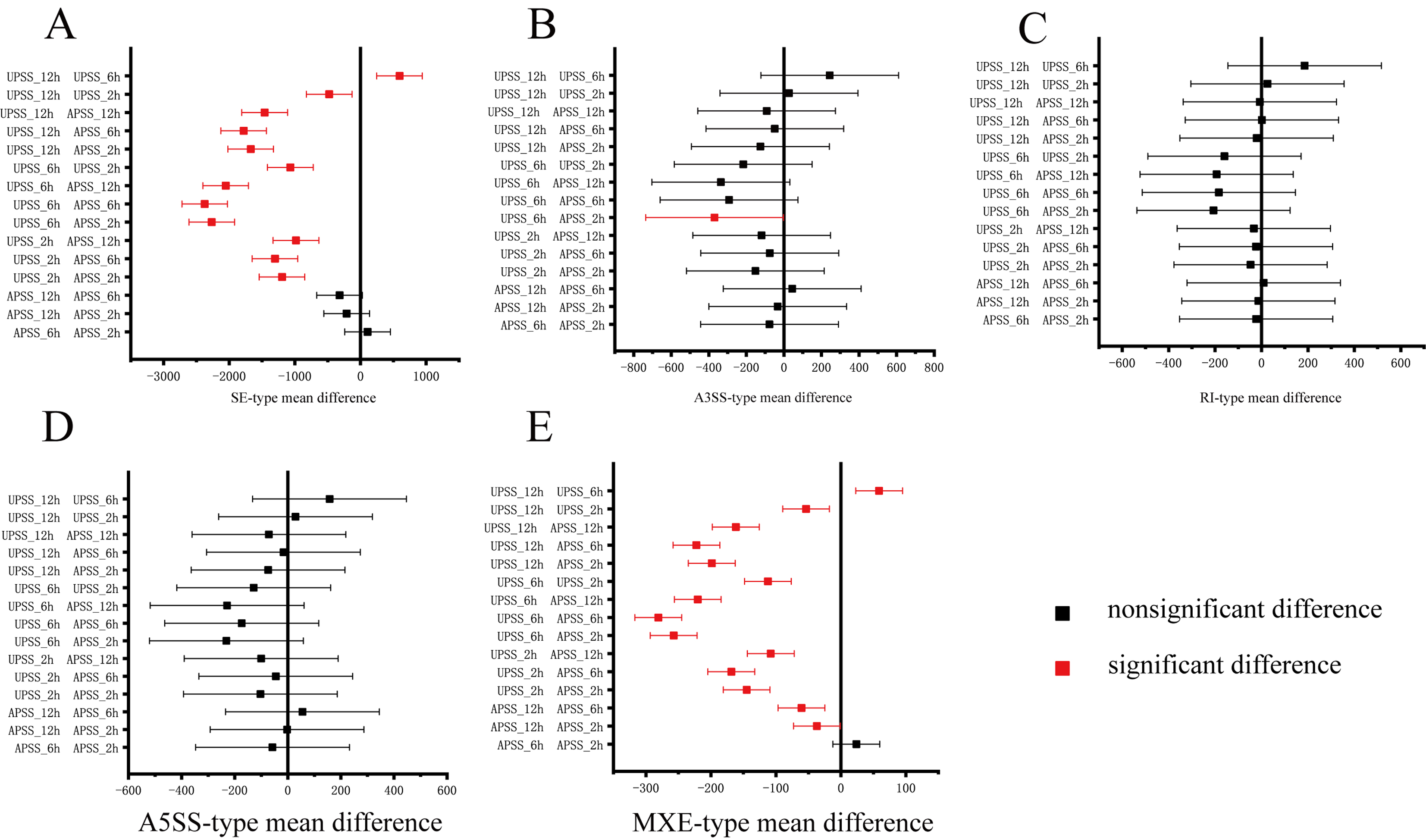

Supplement: Supplementary file 5 [file Image2.JPEG]

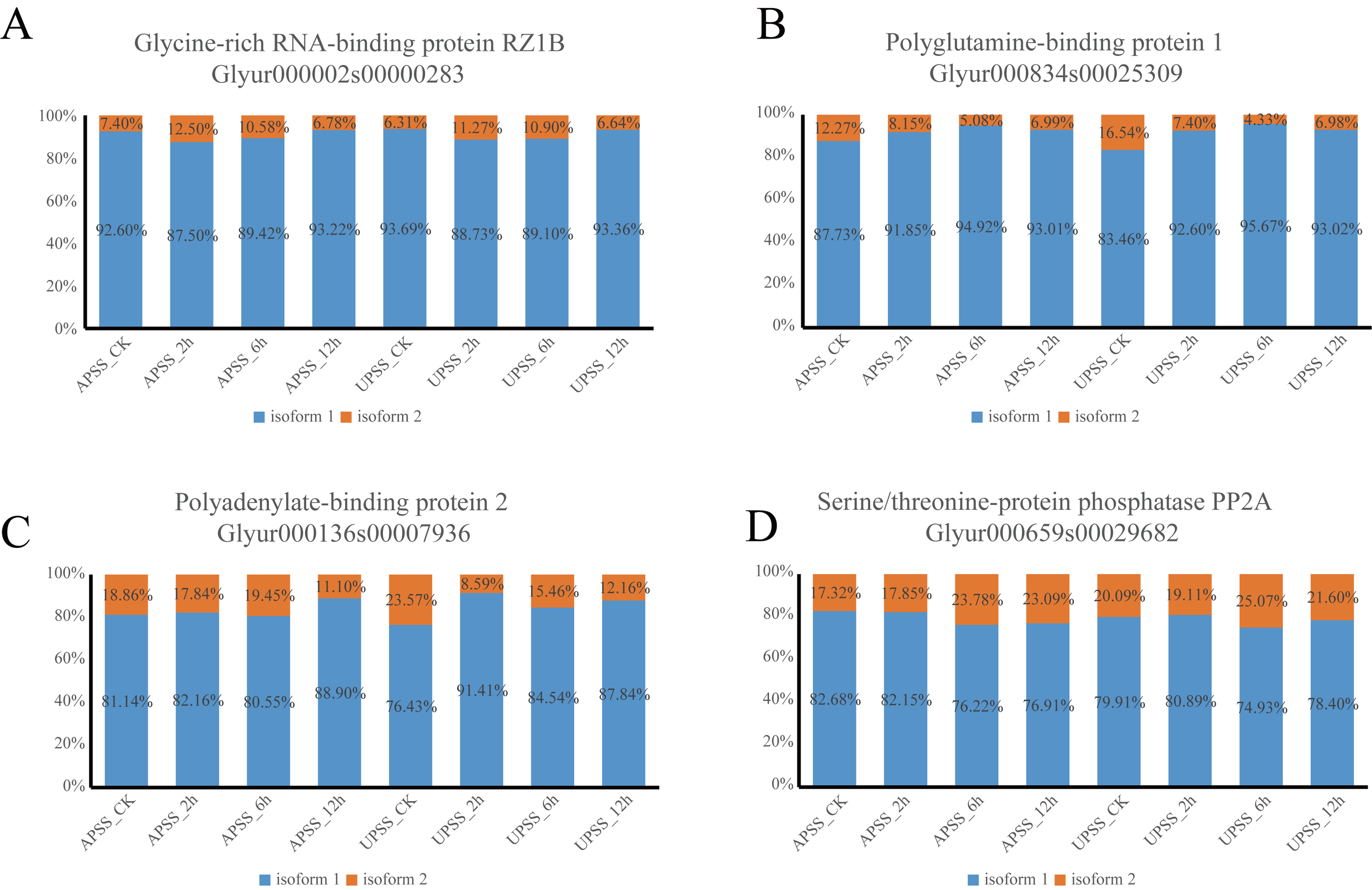

Supplement: Supplementary file 6 [file Image5.JPEG]
